# Resistin in Tissue Remodeling and Fibrosis: A New Frontier

**DOI:** 10.3390/biom16081108

**Published:** 2026-07-29

**Authors:** Barkin Ergun, Mehreen Ahmed, Djamel Lebeche

**Affiliations:** 1 Department of Physiology, College of Medicine, The University of Tennessee Health Science Center, Nash Building, 894 Union Ave, Memphis, TN 38163, USA; bergun@uthsc.edu (B.E.);; 2 Department of Medicine, College of Medicine, The University of Tennessee Health Science Center, Nash Building, 894 Union Ave, Memphis, TN 38163, USA; 3 Neuroscience Institute, College of Medicine, The University of Tennessee Health Science Center, Nash Building, 894 Union Ave, Memphis, TN 38163, USA; 4 Department of Pharmaceutical Sciences, College of Pharmacy, The University of Tennessee Health Science Center, Nash Building, 894 Union Ave, Memphis, TN 38163, USA

**Keywords:** resistin, fibrosis, adipokine, heart failure, liver disease, kidney disease, chronic inflammation

## Abstract

Initially identified as a hormone linking obesity to insulin resistance, resistin is now recognized as a pleiotropic mediator whose cellular sources and biological functions differ substantially between humans and rodents. Beyond its established roles in metabolic dysfunction and inflammation, emerging evidence suggests that resistin may contribute to tissue remodeling and fibrosis in a context-dependent manner. This review critically synthesizes mechanistic, translational, and clinical evidence across the heart, liver, lung, and kidney. Reported interactions with candidate receptors or binding partners, including adenylyl cyclase-associated protein 1 (CAP1) and Toll-like receptor 4 (TLR4), link resistin-associated signaling to inflammatory, oxidative-stress, and profibrotic pathways that can influence fibroblast activation, hepatic stellate cell responses, extracellular matrix production, and structural tissue remodeling. However, the strength and nature of the available evidence differ markedly among organ systems. Direct profibrotic effects are most strongly supported in cardiac experimental models and selected hepatic systems, whereas pulmonary mechanistic evidence is derived largely from studies of other RELM/FIZZ family members, particularly RELMα/FIZZ1 and RELMβ/FIZZ2, rather than human resistin itself, and renal evidence remains predominantly associative. We, therefore, propose a mechanistic paradigm shift that expands, rather than replaces, the established inflammatory role of resistin. Within this framework, the “fibrotic switch” is presented as a unifying hypothesis whereby persistent resistin-associated signaling may couple chronic inflammatory and metabolic stress to progressive fibrogenic remodeling, requiring further organ-, species-, and cell-specific validation. Defining the relevant cellular sources, receptors, and causal pathways will be essential for evaluating resistin as a biomarker and potential therapeutic target in fibrotic disease.

## 1. Introduction

Fibrosis, the pathological deposition of scar tissue, is the common consequence of many chronic inflammatory and metabolic diseases, leading to organ failure and significant mortality. The pathogenesis of fibrosis involves the persistent activation of effector cells—such as hepatic stellate cells (HSCs), fibroblasts, and myofibroblasts—resulting in a dysregulation between the synthesis and degradation of extracellular matrix (ECM) components [1,2]. While transforming growth factor-beta (TGF-β) is considered a major profibrotic cytokine [2], the triggers that initiate and sustain the fibrogenic response, in the context of systemic metabolic disease, remain incompletely understood.

Resistin was first described by Steppan et al. as a cysteine-rich hormone expressed and secreted by mouse adipocytes, where it plays a role in adipocyte differentiation by impairing glucose uptake and interfering with insulin action [3]. Initially, research on resistin focused exclusively on mouse models, but subsequent studies have demonstrated that resistin and resistin-like molecules are also expressed in various human tissues, including the brain, lung, heart, cerebrospinal fluid, placenta, digestive system, reproductive system, kidney and endocrine glands [4,5,6,7,8,9,10].

Resistin is encoded by the *Retn/RETN* gene, which is located on chromosome 8a1 in mice and 19p13.2 in humans [11,12]. Elevated resistin concentrations have been associated with a range of clinical conditions, including type 2 diabetes mellitus (T2DM), atherosclerosis, inflammation, obesity, heart failure, sepsis, rheumatological disorders, cardiovascular disease, and cancer [13,14,15,16,17,18,19,20,21,22]. In mice, resistin directly impairs insulin-stimulated glucose uptake in the liver, skeletal muscle, and cardiomyocytes, promoting systemic insulin resistance [23]. Elevated resistin disrupts glucose homeostasis by inhibiting adenosine monophosphate-activated protein kinase (AMPK), which contributes to dysregulated hepatic insulin signaling and altered glucose metabolism [24,25]. It also disturbs hepatic glycogen regulation by reducing insulin receptor expression and glycogen synthase activity while increasing glycogen phosphorylase activity, thereby decreasing glycogen storage through impaired glycogenesis and enhanced glycogenolysis in primary rat hepatocytes [26]. Treatment with peroxisome proliferator-activated receptor gamma (PPAR-γ) agonist thiazolidinediones (TZDs) has been shown to lower elevated blood glucose partly by reducing serum resistin levels [3,27,28]. Beyond peripheral tissues, resistin may also act within the endocrine pancreas. Sassek et al. reported that resistin is expressed in rat pancreatic islets—primarily in glucagon-positive α-cells—and that exogenous resistin suppresses insulin secretion and modulates glucagon release in a glucose-dependent manner, suggesting a local role in pancreatic hormone regulation [29].

In humans, by contrast, resistin is expressed predominantly in monocytes/macrophages rather than adipocytes and functions primarily as a pro-inflammatory cytokine associated with chronic low-grade inflammation in obesity, type 2 diabetes, and cardiometabolic disease [30,31]. Circulating human resistin levels correlate with insulin resistance, incident type 2 diabetes, and cardiovascular disease, and experimental data implicate resistin in endothelial dysfunction, vascular smooth muscle proliferation, foam-cell formation, and arterial inflammation, thereby contributing to atherosclerosis and adverse cardiovascular outcomes [30,31,32]. Beyond metabolism and vascular biology, both human and mouse resistin are now recognized as host-defense peptides with broad antimicrobial activity [33], the ability to modulate innate immune responses, and a role in limiting excessive inflammation triggered by microbial products, highlighting a dual function at the interface of immunity, inflammation, and energy metabolism [30,33,34].

Previous work on resistin has focused predominantly on its effects on inflammatory and metabolic signaling. Emerging evidence now supports an additional role in which resistin can act directly on fibroblasts, hepatic stellate cells, and other stromal populations to promote myofibroblast activation and extracellular matrix remodeling in several experimental settings. This review therefore advances a paradigm shift that positions resistin not only as an established inflammatory mediator, but also as an emerging regulator of fibrogenic cell behavior. In this framework, inflammatory amplification and direct stromal activation are complementary rather than competing mechanisms through which resistin may contribute to progressive tissue remodeling. We aim to synthesize and critically evaluate evidence across organ systems, while distinguishing direct stromal effects from inflammation-dependent and associative findings. Our objective is to provide a cohesive mechanistic framework that positions resistin as a potential link between chronic inflammatory and metabolic stress and fibrotic remodeling, while identifying the conceptual and translational gaps that remain to be addressed.

## 2. Literature Search and Evidence Selection

This narrative review was developed through targeted searches of PubMed and backward screening of the reference lists of relevant primary studies and review articles. The search covered publications from the initial identification of resistin in 2001 through 16 July 2026. Initial searches combined “resistin” or “*RETN*” with “fibrosis,” “fibrogenesis,” “tissue remodeling,” “extracellular matrix,” “fibroblast,” and “myofibroblast.” Organ-specific searches were then performed using combinations such as “resistin and cardiac fibrosis,” “resistin and liver fibrosis,” “resistin and pulmonary fibrosis,” and “resistin and kidney fibrosis.” Additional searches examined candidate receptors, intracellular signaling pathways, and therapeutic approaches relevant to resistin-associated remodeling. A focused update search was also performed for studies published from 2024 through July 2026.

Direct resistin–fibrosis evidence was not equally available across organ systems. When the initial organ-specific searches identified few or no studies directly examining resistin-mediated fibrosis, the search was expanded to closely related outcomes to characterize the available evidence and its limitations. In the renal literature, studies evaluating circulating resistin in relation to chronic kidney disease severity, renal function, albuminuria, inflammation, endothelial injury, and cardiovascular risk were therefore included as associative clinical evidence rather than as direct evidence of renal fibrogenesis. In the pulmonary literature, direct evidence involving human resistin was limited; consequently, searches were expanded to individual resistin-like molecules, including RELMα/FIZZ1, RELMβ/FIZZ2, and RELMγ. These studies were included to evaluate potential family-level mechanisms of pulmonary remodeling but were not interpreted as demonstrating identical profibrotic actions of human resistin.

Peer-reviewed original studies involving human participants or tissues, animal models, and relevant in vitro systems were considered. Primary studies were prioritized for mechanistic and organ-specific conclusions, whereas selected review articles were used to provide biological context and identify additional original reports. Studies were evaluated according to the specific resistin-family member examined, species, cellular source, experimental model, target organ, and type of outcome. Evidence derived from genetic manipulation, direct protein exposure, or receptor and pathway perturbation accompanied by fibrotic or structural-remodeling outcomes was interpreted as mechanistic evidence. Human tissue observations and associations between circulating resistin, disease severity, organ function, or clinical outcomes were categorized separately as translational or biomarker evidence and were not interpreted as causal in the absence of experimental support. Because this article is a narrative review rather than a systematic review or meta-analysis, no formal risk-of-bias assessment or quantitative evidence-pooling procedure was performed.

## 3. Resistin and Resistin-like Family

The resistin-like molecule/found in inflammatory zone (RELM/FIZZ) family comprises a group of cysteine-rich secreted proteins with species-specific members. In rodents, this family includes Resistin/FIZZ3 (*Retn*), RELMα/FIZZ1 (*Retnlα*), RELMβ/FIZZ2 (*Retnlβ*), and RELMγ (*Retnlγ*), whereas only *RETN* and *RETNLβ* have been clearly identified in humans [35,36]. Despite sharing structural homology, RELM/FIZZ family members differ substantially in their cellular sources, tissue distribution, and biological functions across species [3,35,36].

In rodents, resistin is primarily expressed by adipocytes and is closely linked to insulin resistance and metabolic dysfunction [3,24,25,28]. In contrast, in humans, resistin is predominantly produced by immune cells such as monocytes, macrophages, and neutrophils, and is characterized more as a pro-inflammatory adipokine than as a classical metabolic hormone [3,36,37,38,39]. Other members of the resistin family display more tissue-specific expression: RELMα/FIZZ1 has been linked to pulmonary inflammation and fibrotic remodeling in rodents [40,41,42], whereas human RELMβ/FIZZ2 is highly expressed in the intestine and can also be detected in lung [35,36]. Under hypoxic conditions, RELMβ is induced by hypoxia and promotes proliferation of human lung epithelial, pulmonary artery smooth muscle, and fibroblast-derived cells, suggesting a potential role in pulmonary vascular remodeling and hypoxia-associated fibrotic lung disease. RELMγ remains comparatively less characterized [3,35,36].

## 4. Differences Between Mouse and Human Resistin

Differences between mouse and human resistin can be categorized into molecular structure, gene localization, and signaling (Table 1). Mouse resistin is an 11 kDa protein encoded by the *Retn* gene on chromosome 8a1. It is secreted as a polypeptide precursor that undergoes post-translational cleavage, forming disulfide bond-dependent oligomers, including trimers (the most active form) and hexamers [3,11,27,33,43,44]. In contrast, human resistin is a 12.5 kDa protein encoded by the *RETN* gene on chromosome 19p13.2. Upon secretion, it primarily consists of an alpha-helical coiled-coil domain and a cysteine residue at position 6, which is crucial for its oligomerization [3,11,27,33,43]. Mouse resistin is mainly expressed in white adipose tissue, whereas human resistin is predominantly expressed in immune cells such as macrophages, monocytes, and neutrophils [10,38,45]. Additionally, human resistin contains an arginine-glycine-aspartic acid (RGD) motif that is absent in mouse resistin. Overall, these differences result in approximately 59% sequence similarity between mouse and human resistin [3,27,46].

These species-specific cellular sources are likely to produce distinct patterns of tissue-associated resistin expression and localization. Consistent with this distinction, resistin mRNA expression in human adipose tissue has been reported to be approximately 250-fold lower than that in mouse adipose tissue [11]; however, this comparison concerns mRNA expression rather than direct tissue protein concentrations. At the protein level, systematic immunohistochemical analysis of normal human tissues showed that human resistin is broadly distributed and principally localized in cytoplasmic granules of macrophages or macrophage-like cells scattered within tissue interstitia, with additional extracellular staining [9]. Human *RETN* expression is also enriched in myeloid and immune cells [37,38], and neutrophils can store and release resistin in response to immune inflammatory stimulation [39]. Thus, adipocyte production is likely to contribute more prominently to tissue-associated resistin in rodents, whereas in humans the local distribution may be shaped more strongly by the presence and recruitment of resistin-producing immune cells. These species-dependent differences should be considered when interpreting tissue resistin measurements and extrapolating findings from rodent models to human fibrosis.

### Translational Considerations and Limitations of Experimental Models

These species differences have important implications for fibrosis research. Conventional murine *Retn* models predominantly reflect adipocyte-derived resistin biology and therefore do not fully reproduce the immune-cell-dominant pattern of human *RETN* expression. In addition, RELMα/FIZZ1, RELMβ/FIZZ2, and RELMγ should not be considered interchangeable with either murine or human resistin, because these family members differ in their cellular sources, tissue distribution, and biological functions [35,36]. Findings involving RELM proteins may therefore identify family-level or potentially shared biological mechanisms, but they do not constitute direct evidence that human resistin produces the same fibrotic response.

Mouse models expressing human resistin can partially address this translational gap. Qatanani et al. generated *Retn*-deficient mice expressing human *RETN* predominantly in the monocyte/macrophage lineage, thereby reproducing a cellular source more similar to that observed in humans [38]. This model demonstrated that macrophage-derived human resistin can promote adipose tissue inflammation and metabolic dysfunction in vivo, although fibrosis was not directly investigated. In a separate study using humanized experimental models, Yang et al. showed that endocannabinoid signaling increased resistin expression in CB1R-positive human peripheral blood mononuclear cells through the p38–Sp1 pathway. In humanized NOG and humanized resistin mouse models, high-fat feeding promoted the recruitment of resistin-producing cells to metabolic tissues, including adipose tissue, heart, skeletal muscle, and liver, with accompanying inflammation and insulin resistance; these responses were attenuated by CB1R blockade or resistin deficiency [47]. In a cardiac- specific transgenic model, Lin et al. showed that cardiac-specific human resistin overexpression was sufficient to induce cardiac dysfunction and maladaptive remodeling in the setting of pulmonary hypertension [48]. Although these models provide valuable evidence that human resistin remains biologically active in vivo, they retain a murine immune, receptor, and tissue environment and may involve tissue-specific or nonphysiological expression. Human-*RETN*-expressing mouse models therefore improve translational relevance but do not fully eliminate species-related uncertainty. Future studies integrating these models with primary human cells, human tissue analyses, and longitudinal clinical data will be required to determine which resistin-associated fibrogenic mechanisms are conserved in human disease.

## 5. Molecular Pathways of Resistin

Although the mechanism of resistin action in humans has not been fully elucidated, accumulating evidence supports a crucial role for resistin in modulating inflammation, immune responses, and cellular stress. Rather than acting through a single canonical receptor or linear cascade, resistin appears to activate or bind to multiple receptors and downstream pathways, thereby shaping inflammatory, metabolic, and stress-related cellular responses.

### 5.1. Receptor Engagement and Signal Initiation

**Figure 1:**
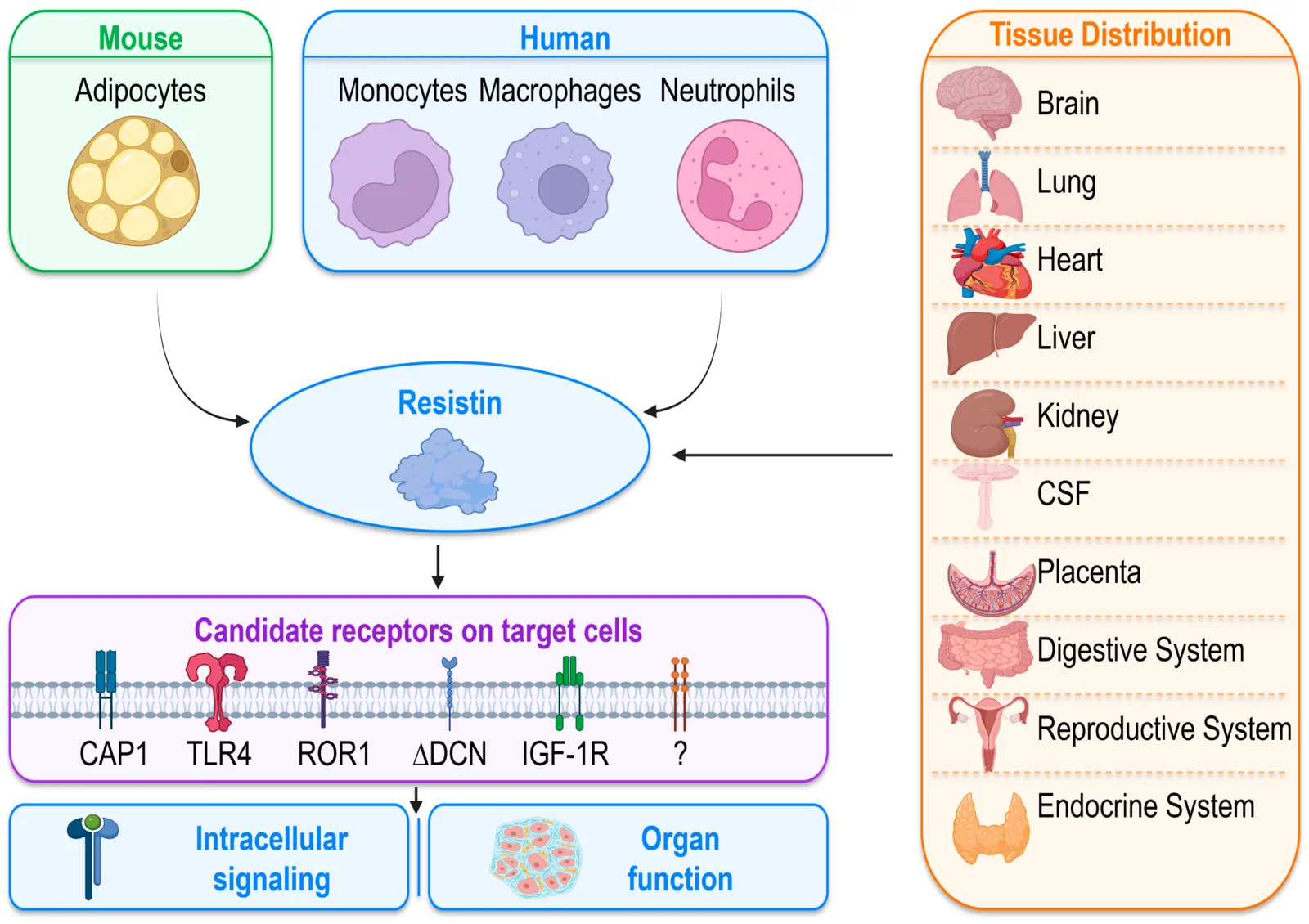
Cellular sources, reported distribution, and candidate receptor interactions of resistin. Resistin displays distinct patterns of cellular origin in mice and humans. In mice, it is mainly produced by adipocytes, whereas in humans it is largely derived from immune cells, including monocytes, macrophages, and neutrophils. Resistin has also been reported in multiple tissues and biological compartments, including the brain, lung, heart, liver, kidney, cerebrospinal fluid, placenta, digestive system, reproductive system, and endocrine system. After secretion, resistin interacts with several proposed receptors on target cells, including CAP1, TLR4, ROR1, ΔDCN, and IGF-1R, triggering intracellular signaling networks that regulate organ function. Since an exact resistin receptor has not yet been conclusively established, the question mark denotes the possibility that additional, as-yet-unidentified receptor(s) or binding partner(s) may also contribute to resistin signaling. Created in BioRender. Ergun, B. (2026) https://BioRender.com/mvrln1u.

Although the *exact* receptor for resistin remains unresolved, several functional interactions with respective candidate receptors or binding partners—including TLR4, CAP1, Receptor tyrosine kinase-like orphan receptor 1 (ROR1), Delta-decorin (ΔDCN), and Insulin-like growth factor 1 receptor (IGF-1R)—have been reported [49,50,51,52,53]. Among these candidates, CAP1 and TLR4 are the most extensively characterized in human immune cells, while ΔDCN and ROR1 appear to have more specific effects [49,50,52,53] (Figure 1).

Among the proposed receptors for resistin, CAP1 has been identified as a functional receptor in human monocytes and appears to play an important role in resistin-induced inflammatory signaling. Resistin binding to CAP1 increases intracellular cyclic AMP (cAMP) levels, activates protein kinase A (PKA), and promotes nuclear factor kappa B (NF-κB)–dependent expression of pro-inflammatory cytokines, including Interleukin-6 (IL-6), Interleukin-1 beta (IL-1β), and tumor necrosis factor alpha (TNF-α) [53]. In mouse liver cells, CAP1 has also been reported to mediate resistin-induced expression of genes related to insulin resistance, inflammation, and apoptosis [54]. More recently, CAP1-dependent resistin signaling was demonstrated in human fibroblast-like synoviocytes. Resistin activated a CAP1/PKA/CREB pathway that increased CPT1A-dependent fatty acid oxidation and promoted inflammatory and catabolic cellular responses. Although this study examined metabolic syndrome-associated osteoarthritis rather than fibrosis, it extends CAP1-mediated resistin signaling to a human stromal-like cell population and further illustrates its cell-context-dependent effects [55].

TLR4 has been implicated as another candidate receptor mediating resistin signaling. In a human epithelial kidney cell line, resistin–TLR4 interaction was reported to mediate pro-inflammatory effects [49]. In the rat hypothalamus, this interaction activated downstream signaling through myeloid differentiation primary response 88 (MyD88) and toll/interleukin-1 receptor domain-containing adaptor protein (TIRAP), leading to stimulation of c-Jun N-terminal kinase (JNK) and p38 mitogen-activated protein kinase (p38) pathways [56]. Similarly, in porcine alveolar macrophages, resistin activated the TLR4/NF-κB pathway [57]. Together, these findings suggest that resistin can engage CAP1- and TLR4-dependent pathways to regulate inflammatory gene expression, metabolic stress signaling, and apoptosis in a context-dependent manner.

Resistin’s relationship with Δ-decorin has gained attention because an isoform of decorin has been identified as a functional resistin receptor in adipocyte progenitors. Evidence indicates that Δ-decorin can bind resistin and regulate white adipose tissue expansion, suggesting that decorin family members may act as modulators of resistin-driven adipogenesis and metabolic signaling [50]. In addition, decorin itself is known to regulate multiple receptor tyrosine kinases, including IGF-1R, by modulating their activity and downstream signaling pathways [58,59]. Although direct binding between resistin and IGF-1R has not been conclusively demonstrated, the ability of decorin to influence IGF-1R signaling raises the possibility of indirect or context-dependent interactions within tissues where both molecules are expressed. In another study conducted in rheumatoid arthritis synovial fibroblasts, resistin stimulation was shown to modulate IGF-1R expression, but with no evidence of direct interaction [51]. IGF-1R may be better interpreted as part of a broader signaling network that can be modulated during resistin-associated cellular responses.

The interaction between resistin and ROR1 is supported by more direct experimental evidence. Mouse resistin has been shown to bind specific extracellular domains of ROR1, inhibiting its phosphorylation and altering downstream extracellular signal-regulated kinase 1/2 (ERK1/2) signaling. This interaction affects expression of suppressor of cytokine signaling 3 (SOCS3), glucose transporter type 4 (GLUT4), and glucose transporter type 1 (GLUT1), ultimately modulating glucose uptake and promoting adipogenesis in 3T3-L1 cells [52]. These findings position ROR1 as a plausible resistin receptor in certain metabolic contexts, particularly in adipocyte biology.

Together, these studies suggest that resistin may act through a network of receptor or co-receptor interactions—Δ-decorin potentially serving as a binding partner in progenitor cells, ROR1 functioning as a signaling receptor in adipogenesis, and IGF-1R pathways being indirectly influenced through decorin-mediated regulation (Figure 1). While none of these candidates have been definitively established as the bona fide resistin receptor, they collectively highlight the complexity of resistin signaling and its integration into broader metabolic and inflammatory pathways.

### 5.2. Early Intracellular Signaling and Pathway Convergence

After receptor binding, resistin rapidly triggers downstream intracellular signaling through several pathways, including the activation of phosphoinositide 3-kinase (PI3K)/Protein kinase B (AKT), mitogen-activated protein kinase (MAPK) cascades ERK1/2, p38, and JNK), and adenylate cyclase–cAMP–PKA signaling, as well as calcium-dependent mechanisms involving L-type voltage-sensitive calcium channels and phospholipase C [53,56,60,61,62].

**Figure 2:**
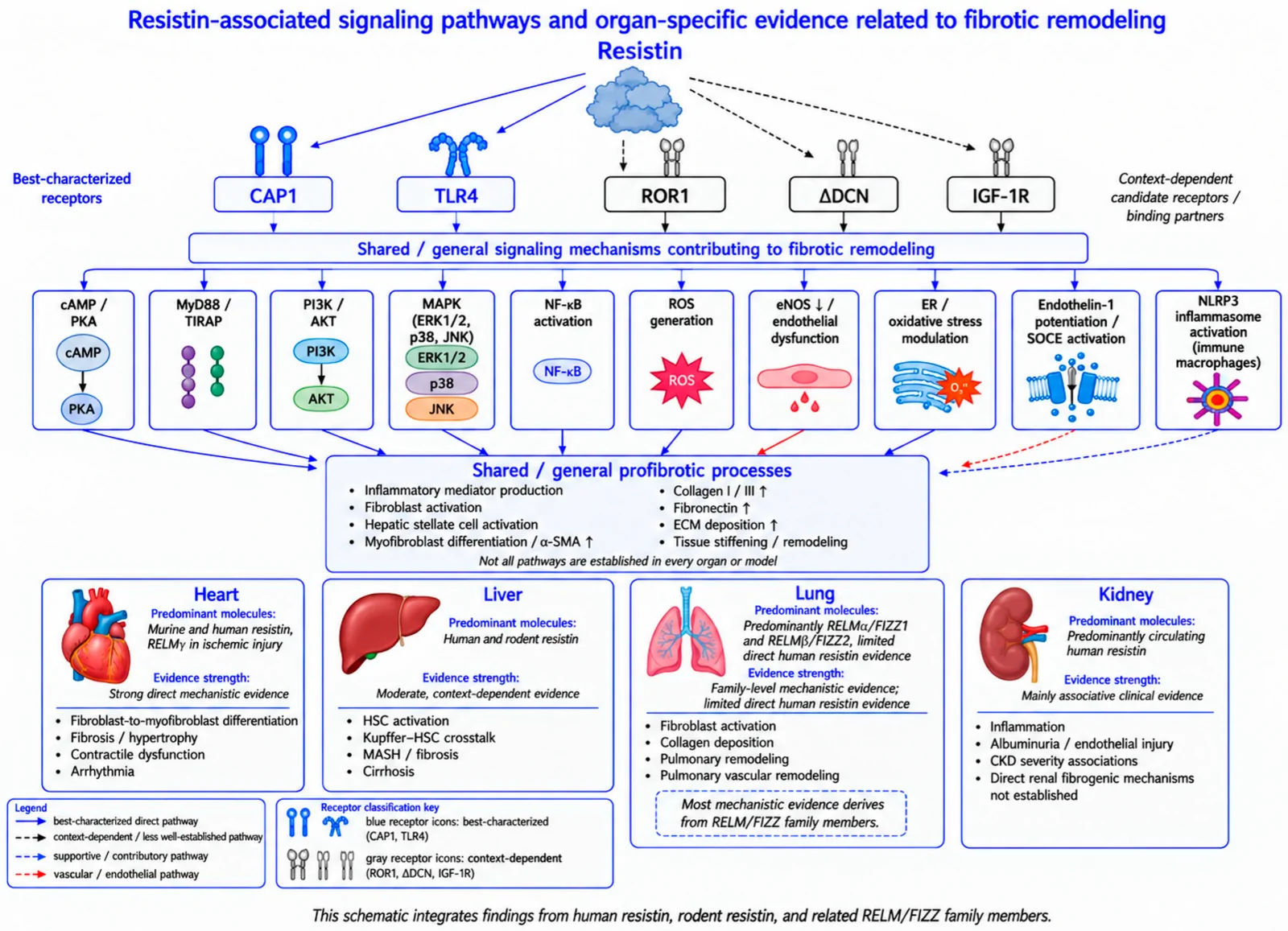
Resistin-associated signaling pathways and organ-specific evidence related to fibrotic remodeling. This schematic summarizes signaling pathways and cellular processes that have been associated with resistin-related fibrotic remodeling across organ systems. CAP1 and TLR4 are shown as the best-characterized receptors, whereas ROR1, ΔDCN, and IGF-1R are presented as context-dependent candidate receptors or binding partners. Downstream pathways reported in different experimental settings include cAMP/PKA, MyD88/TIRAP, PI3K/AKT, MAPK (ERK1/2, p38, JNK), NF-κB activation, ROS generation, endothelial dysfunction, ER/oxidative stress modulation, endothelin-1 potentiation/SOCE activation, and NLRP3 inflammasome activation. These pathways may converge on shared profibrotic processes, including inflammatory mediator production, fibroblast activation, hepatic stellate cell activation, myofibroblast differentiation, extracellular matrix deposition, collagen accumulation, and tissue stiffening/remodeling. The lower panels summarize the predominant molecules and relative evidence base across organs. Cardiac evidence includes murine and human resistin, with additional evidence involving RELMγ in ischemic injury, and provides the strongest direct mechanistic support for profibrotic remodeling. Hepatic evidence involves both human and rodent resistin and supports context-dependent profibrotic activity. In the lung, much of the mechanistic evidence derives from RELMα/FIZZ1 and RELMβ/FIZZ2 rather than human resistin itself; therefore, pulmonary findings should not be interpreted as direct proof of identical profibrotic actions of human resistin. In the kidney, the available evidence is predominantly associative and clinical, with direct resistin-mediated fibrogenic mechanisms not yet established. This figure integrates findings from human resistin, rodent resistin, and related RELM/FIZZ family members and should therefore be interpreted as a conceptual summary of the currently available evidence rather than a universally established pathway operating in every organ or model. Created in BioRender. Ergun, B. (2026) https://BioRender.com/mvrln1u. Image generated with licensed copy of BioRender.

A major consequence of activating these pathways is robust NF-κB activation, which promotes the expression of pro-inflammatory cytokines, chemokines, and adhesion molecules, thereby amplifying cellular inflammation. Concurrently, activated MAPK signaling contributes to cell proliferation, oxidative stress responses, and transcriptional reprogramming [63,64,65,66]. Recent evidence identifies the NLRP3 inflammasome as an additional downstream pathway of human resistin. In human macrophages, resistin induced HMGB1-dependent NF-κB priming of NLRP3 pathway components and bound to Bruton’s tyrosine kinase (BTK), promoting BTK autophosphorylation, NLRP3 phosphorylation, inflammasome assembly, and subsequent IL-1β and IL-18 release [67]. (Figure 2).

### 5.3. Oxidative and Endoplasmic Reticulum Stress Regulation of Resistin

Resistin signaling is closely associated with oxidative stress. In human coronary artery endothelial cells, resistin increases reactive oxygen species production, activates p38/JNK MAPK signaling, and suppresses endothelial nitric oxide synthase (eNOS) expression and nitric oxide bioavailability [68]. This redox imbalance may further contribute to endothelial dysfunction and inflammatory vascular remodeling [68,69]. Under conditions of endoplasmic reticulum (ER) stress, impaired secretion of human resistin leads to its intracellular accumulation within the ER, where it may exert a chaperone-like function by interacting with misfolded proteins, facilitating protein folding and quality-control processes, and thereby contributing to cellular adaptation and protection against stress-induced apoptosis [70,71] (Figure 2).

## 6. Resistin in Fibrotic Diseases

Disruptions in organ homeostasis can lead to diseases such as obesity, cardiac failure, insulin resistance, diabetes, non-alcoholic fatty liver disease (NAFLD), metabolic dysfunction-associated steatohepatitis (MASH), dyslipidemia, and cancer [72,73,74,75,76,77]. Although it remains unclear whether elevated resistin primarily reflects disease activity or contributes to disease progression, normal circulating resistin concentrations, typically reported in the range of 7–22 ng/mL, are increased in several metabolic and inflammatory conditions [14,69,78]. Consistent with these observations, experimental evidence further suggests that resistin upregulation may have tissue-specific functional consequences, including the promotion of fibrogenic responses through hepatic stellate cell activation and fibroblast-to-myofibroblast differentiation [79,80] (Figure 1).

### 6.1. Cardiac Fibrosis and Remodeling: Experimental and Clinical Evidence

Multiple studies have demonstrated a strong association between resistin and cardiac fibrosis. We have previously reported that cardiac-specific overexpression of resistin in rats induces a diabetic cardiomyopathy phenotype characterized by myocardial fibrosis and cardiac contractile dysfunction through systolic impairment and adverse remodeling [81]. These effects are mediated by oxidative stress, likely driven by nicotinamide adenine dinucleotide phosphate (NADPH) oxidase and by TNF-α/NF-κB–dependent inflammatory signaling, ultimately leading to cardiac fibrosis, apoptosis, and disrupted calcium homeostasis through a reduced sarco/endoplasmic reticulum Ca^2+^-ATPase 2a (SERCA2a)/phospholamban ratio [81]. Furthermore, we showed in another study that genetic deletion of resistin attenuates pressure overload–induced cardiac fibrosis, as evidenced by reduced expression of fibrotic markers including collagen type I alpha 1 (COL1A1), fibronectin, cellular communication network factor 2 (CCN2), and lysyl hydroxylase (LOX) [82]. Conversely, resistin overexpression exacerbated fibrotic remodeling [82]. Resistin deficiency was associated with increased expression of the DNA repair protein growth arrest and DNA damage-inducible alpha (Gadd45a), whereas elevated resistin levels suppressed Gadd45a via miR-148b-3p, thereby activating the DNA damage response and promoting cardiomyocyte apoptosis and fibrosis [82].

Resistin has also been shown to potentiate and prolong the vasoconstrictive effects of endothelin-1 by activating store-operated calcium entry (SOCE) in vascular smooth muscle cells [83]. In addition to its vasoconstrictive properties, resistin has been implicated in cardiac hypertrophic remodeling. In H9C2 cardiomyoblasts, resistin induces hypertrophic responses characterized by increased cell surface area, enhanced protein synthesis, and upregulation of brain natriuretic peptide (BNP) and beta-myosin heavy chain (β-MHC) [84]. A subsequent study further showed that resistin increases atrial natriuretic factor (ANF), BNP, and β-MHC expression, together with activation of ERK and TLR4/MyD88/NF-κB signaling; these effects were attenuated by omentin, suggesting a potential protective role against resistin-induced cardiomyoblast hypertrophy [85]. Furthermore, resistin promotes fibroblast-to-myofibroblast differentiation. We demonstrated that resistin upregulates fibrotic markers such as alpha-smooth muscle actin (α-SMA), COL1A1, connective tissue growth factor (CTGF), fibronectin, and LOX in cardiac tissue. This effect is mediated through activation of the janus kinase 2 (JAK2)/STAT3 and JNK/c-Jun pathways, leading to phosphorylation and nuclear translocation of STAT3 and c-Jun in the absence of smad family member 3 (Smad3) activation, indicating that this signaling occurs independently of the canonical TGF-β pathway [79].

Resistin-associated inflammatory responses may disrupt cardiac Ca^2+^ homeostasis through cytokines such as Interleukin-17(IL-17). Xue et al. revealed that IL-17 downregulates SERCA2a and the L-type calcium channel Cav1.2 through NF-κB activation, leading to impaired Ca^2+^ handling and contractility [86]. Moreover, IL-17–deficient hearts exhibit reduced susceptibility to cardiac fibrosis, consistent with the concept that calcium dysregulation contributes to inflammation-driven fibrotic remodeling in the heart [86]. In cardiomyocytes, TNF-α has similarly been shown to inhibit SERCA2a activity via NF-κB–dependent binding to the SERCA2a promoter, resulting in impaired calcium reuptake and diastolic dysfunction [87].

Circulating resistin levels also rise during ischemia–reperfusion injury and correlate with myocardial damage and oxidative stress, suggesting potential utility as a biomarker in this setting [88]. Recent evidence showed that the resistin family contributes to ischemic cardiac injury through mechanisms beyond classical inflammatory signaling. Following myocardial infarction, neutrophil-derived resistin-like molecule γ (RELMγ/*Retnlg*) accumulates in ischemic myocardium and directly damages stressed cardiomyocyte membranes, leading to calcium influx and ventricular arrhythmias [89]. Notably, human resistin shows similar membrane-disruptive effects, indicating that resistin-family proteins may worsen ischemic tissue injury not only through inflammatory and fibrotic pathways but also through direct membrane damage [89]. In a separate HFpEF context, Liu et al. identified *Relmg* as a fibrosis-associated candidate in mice subjected to transverse aortic constriction and deoxycorticosterone acetate treatment. Myocardial fibrosis was accompanied by increased *Relmg* and reduced *Adcy1* expression [90]. However, because the study relied on fibrosis-related transcriptomic screening and expression validation rather than direct genetic or pharmacological perturbation of RELMγ, these findings support an association with HFpEF-related fibrosis but do not establish a causal profibrotic role [90].

Cardiac hypertrophy can be broadly categorized into pressure-overload and volume-overload forms, each associated with distinct structural and remodeling profiles [91]. Chemaly et al. demonstrated that pressure-overload hypertrophy is characterized by pronounced fibrosis, reduced capillary density, increased wall stress, and impaired oxygen supply–demand balance, whereas volume-overload hypertrophy exhibits minimal fibrosis despite comparable ventricular remodeling [92]. Myocardial resistin expression was markedly increased in pressure-overload hypertrophy and locally elevated in chronically ischemic/infarcted myocardium, with resistin-induced CTGF expression in adult cardiomyocytes providing a mechanistic link to profibrotic signaling in ischemia-associated cardiac fibrosis [92].

Additionally, Muse et al. examined circulating resistin levels in individuals without baseline cardiovascular disease within the Multi-Ethnic Study of Atherosclerosis (MESA) cohort. They found that higher resistin concentrations were associated with a stepwise increase in the incidence of heart failure, coronary heart disease, and overall cardiovascular events [93]. Beyond its effects on endothelial activation and foam-cell formation, resistin may contribute to atherosclerotic plaque remodeling by stimulating vascular smooth muscle cell (VSMC) proliferation and the production of fibrous-cap components. However, its pro-inflammatory effects may simultaneously favor features associated with plaque instability. Thus, the overall influence of resistin on plaque stability is likely to depend on disease stage, cellular context, and the balance between matrix production and inflammatory injury [94,95].

Although the mechanistic studies discussed so far support a direct profibrotic role for resistin in cardiac fibrogenesis, findings from the MESA cohort suggest a more complex relationship. In this cohort, elevated circulating resistin levels were associated with an increased risk of heart failure with reduced ejection fraction (HFrEF), yet no significant association was observed between resistin concentrations and myocardial fibrosis [17]. Taken together, the cardiac literature provides the strongest support for a direct profibrotic role of resistin among the organ systems considered in this review. Genetic manipulation, cardiac overexpression, and cell-based studies demonstrate effects on fibroblast differentiation, profibrotic signaling, apoptosis, and adverse remodeling [79,81,82,92]. Nevertheless, the clinical evidence remains less definitive: circulating resistin is associated with cardiovascular events and heart failure risk but has not consistently correlated with myocardial fibrosis itself [17,93]. Thus, cardiac data support a mechanistic contribution of resistin to fibrotic remodeling, while its value as a circulating marker of myocardial fibrosis requires further validation.

### 6.2. Liver Fibrosis: Mechanistic and Clinical Evidence

Liver fibrosis is a dynamic wound-healing response to chronic hepatic injury, characterized by excessive extracellular matrix deposition driven by persistent inflammation, hepatic stellate cell activation, and complex interactions among immune, parenchymal, and stromal cells [96]. Emerging evidence suggests that members of the resistin family play important roles in linking inflammatory signaling to fibrogenic remodeling in the liver.

Custovic and Rasic evaluated serum adiponectin and resistin levels in patients with chronic hepatitis B and found that serum resistin concentrations were significantly higher in individuals with more advanced fibrosis, whereas adiponectin levels showed no significant association with fibrosis severity [97]. Bertolani et al. further demonstrated that resistin is markedly upregulated during chronic liver injury: although basal expression is low in healthy liver, resistin levels increase substantially in end-stage liver disease and acute alcoholic hepatitis [60]. Resistin localized predominantly to regions of active inflammation and fibrogenesis, including α-SMA–positive cells and collagen-rich areas [60]. While primary human hepatic stellate cells did not constitutively express resistin, recombinant resistin induced monocyte chemoattractant protein-1 (MCP-1) and Interleukin-8 (IL-8) expression via NF-κB signaling, enhancing monocyte chemotaxis without promoting stellate cell proliferation or collagen I or TGF-β1 expression [60].

In a cross-sectional study of patients with NAFLD, serum resistin levels were significantly higher in NAFLD patients than in healthy controls. However, circulating resistin concentrations did not differ between mild and advanced fibrosis groups, and no association was observed between resistin levels and fibrosis severity [98]. In a pilot study of obese individuals with NAFLD, Zyśk et al. investigated salivary pro-inflammatory adipokines and cytokines as potential noninvasive markers of liver injury. Salivary resistin levels differed across groups stratified by obesity status and hepatic steatosis and showed a positive correlation with steatosis severity measured by controlled attenuation parameters. Higher salivary resistin was also associated with increased serum aminotransferase (ALT) and γ-glutamyl transpeptidase (GGT) levels, supporting its potential utility as a noninvasive biomarker of NAFLD-related hepatic injury [99]. In a high-fat diet–induced NAFLD model, progressive hepatic fibrosis was accompanied by a time-dependent increase in hepatic resistin expression and elevated serum fibrosis markers, including procollagen III, hyaluronic acid, collagen IV, and laminin. In vitro, recombinant resistin increased TGF-β1 and TNF-α expression in HSC-T6 hepatic stellate cells, suggesting a potential profibrotic and proinflammatory role for resistin in NAFLD-associated hepatic fibrosis [100]. Consistent with these findings, Nobili et al. reported that the absolute number of resistin-positive hepatic progenitor cells increased in pediatric NAFLD and correlated with fibrosis severity [101].

With persistent liver injury, fibrosis may progress to cirrhosis. Kakizaki et al. measured fasting plasma resistin concentrations in patients with liver cirrhosis and found significantly elevated levels that increased in parallel with disease severity [102]. Hepatic stellate cells are the principal effector cells of liver fibrosis, driving extracellular matrix production through activation, proliferation, migration, and resistance to apoptosis [80,103]. Dong et al. demonstrated that resistin modulates stellate cell behavior and promotes a more profibrogenic phenotype [80]. Circulating resistin levels were elevated in cholestatic liver injury, although hepatic resistin expression remained unchanged [80]. In vitro, resistin promoted stellate cell proliferation and migration and inhibited apoptosis through IL-6– and MCP-1–dependent mechanisms [80]. Resistin also activated Kupffer cells to increase TGF-β1 production, and factors released from resistin-treated Kupffer cells enhanced collagen I and connective tissue growth factor expression in stellate cells [80].

In patients with MASH, Shen et al. reported significantly higher hepatic resistin mRNA and protein expression compared with simple steatosis and healthy controls [104]. Although circulating resistin levels were elevated in NAFLD and did not distinguish MASH from simple steatosis, hepatic resistin expression showed strong positive associations with lobular inflammation and fibrosis stage, and they further showed that resistin immunoreactivity localized predominantly to perisinusoidal cells, hepatic stellate cells, and Kupffer cells [104]. In another NAFLD cohort, Jamali et al. found that serum resistin concentrations were significantly higher in patients with advanced fibrosis compared with those with mild or no fibrosis, suggesting potential utility as a biomarker for advanced hepatic fibrosis [105].

Conversely, several clinical studies have reported findings that do not support a strong association between resistin and liver fibrosis. In a cross-sectional analysis of adipokines in NAFLD, Jarrar et al. found no significant differences in circulating resistin levels among patients with MASH, simple steatosis, obese controls, and non-obese controls, and resistin was not independently associated with disease severity or fibrosis stage [106]. Similarly, Zou et al. reported no differences in circulating resistin levels among obese children with NAFLD, obese children without hepatic steatosis, and non-obese controls; resistin was not associated with steatosis severity, insulin resistance, or biochemical markers of liver injury [107]. In a case–control study, Jamali et al. found that serum resistin levels did not differ significantly between NAFLD patients and healthy controls and were not consistently associated with steatosis grade or lobular inflammation. Although resistin levels were higher in patients with moderate-to-severe fibrosis, overall analyses did not demonstrate a consistent or independent association with histological liver injury [108]. These inconsistent clinical findings may reflect both biological and methodological heterogeneity. Circulating resistin may not parallel intrahepatic resistin expression; in one study, serum resistin did not distinguish MASH from simple steatosis, whereas hepatic resistin expression correlated with lobular inflammation and fibrosis stage [104]. The available studies also differ in age group, obesity status, cohort composition, study design, and the distribution of histological disease stages [97,98,99,100]. Disease stage may also influence circulating resistin levels, as plasma resistin concentrations have been reported to increase with the severity of liver cirrhosis [102,109]. Moreover, because human resistin is predominantly produced by immune cells [37,38,39], circulating levels may be influenced by systemic and hepatic inflammatory activity [60,104,109]. Thus, inconsistent associations between serum resistin and fibrosis severity do not negate a context-dependent contribution of resistin to hepatic remodeling; rather, they highlight the importance of distinguishing circulating biomarker associations from tissue-level expression and mechanistic evidence.

In patients with liver cirrhosis, circulating resistin levels were significantly elevated compared with healthy controls and increased with advancing disease stage [102]. Hepatic venous resistin concentrations exceeded arterial levels, suggesting active hepatic production. Circulating resistin correlated positively with TNF-α, indicating a link to systemic inflammation, whereas no association was observed with measures of insulin resistance [109]. Overall, the hepatic evidence supports a context-dependent relationship between resistin and fibrotic remodeling. Experimental studies demonstrate effects on hepatic stellate cell behavior, Kupffer cell signaling, inflammatory mediator production, and profibrotic gene expression [60,80,100]. Human tissue studies also link intrahepatic resistin expression with inflammation and fibrosis severity [101,104]. However, studies based on circulating resistin have produced inconsistent results across NAFLD, MASH, and cirrhosis cohorts [97,98,102,105,106,107,108,109]. The liver data therefore support biologically plausible and experimentally demonstrated profibrotic actions, but do not establish serum resistin as a uniform marker of fibrosis across all patient populations.

### 6.3. Pulmonary Fibrosis: Human Resistin and RELM/FIZZ Evidence

Pulmonary fibrosis is a multistage pathological process in which an ongoing inflammatory response progresses into persistent fibroblast activation and excessive extracellular matrix deposition [110]. Throughout this progression, epithelial cells, immune cells, and fibroblasts play central roles. Adipokines—including resistin and resistin-like molecules—have been implicated in linking inflammatory signaling to lung fibrotic remodeling; however, most available evidence is derived from studies of RELM/FIZZ proteins, and direct data on human resistin remain limited [35,36]. Importantly, not all members of this protein family contribute equally to pulmonary fibrosis; some primarily regulate inflammation, whereas others actively participate in fibrotic remodeling [36].

Liu et al. demonstrated that *FIZZ1* is markedly upregulated in a bleomycin-induced rat model of pulmonary fibrosis. *FIZZ1* was among the most strongly induced genes in fibrotic lungs and was localized primarily to alveolar and airway epithelial cells, whereas its expression was not detected in isolated lung fibroblasts [40]. They further showed that epithelial-derived *FIZZ1* directly promotes fibroblast-to-myofibroblast differentiation, increasing α-SMA and type I collagen expression independently of TGF-β signaling [40]. In light of evidence that lipofibroblasts can serve as precursors of activated myofibroblasts during pulmonary fibrosis [111], these findings raise the possibility that epithelial-derived *FIZZ1/RELMα* may also contribute to lipofibroblast-to-myofibroblast conversion. However, this specific lineage transition has not been directly tested in response to *FIZZ1/RELMα* and remains an open question. More recently, *FIZZ1/RELMα* upregulation was associated with lncRNA CBR3-AS1/miR-29 dysregulation and increased TGF-β1, Smad3, and hydroxyproline levels in bleomycin-induced pulmonary fibrosis in rats. Trimetazidine treatment reduced *FIZZ1* expression and attenuated histological and molecular indices of fibrosis [41]. However, because *FIZZ1* was not selectively genetically or pharmacologically manipulated, the study does not establish *FIZZ1* as the sole causal mediator of the treatment response.

Rather than directly examining fibrosis, Lin et al. investigated the role of resistin family members in pulmonary vascular remodeling during early pulmonary hypertension (PH). They reported increased expression of human resistin in macrophage-like cells within lung tissues from patients with idiopathic pulmonary arterial hypertension [112]. In a hypoxia-induced mouse model, *RELMα* promoted pulmonary macrophage accumulation and enhanced high-mobility group box-1 (HMGB1) release through suppression of sirtuin-1 (Sirt1) signaling in macrophages [112]. This, in turn, stimulated pulmonary artery smooth muscle cell proliferation via the receptor for advanced glycation end products (RAGE)/HMGB1 mechanism [112]. Extending these findings to a large clinical cohort, Gao et al. measured serum resistin in 1121 adults with pulmonary arterial hypertension, including patients with idiopathic and systemic sclerosis-associated disease. Higher resistin concentrations were associated with shorter six-minute walking distance, reduced cardiac index, and an increased risk of mortality [113]. These clinical associations are supported by recent mechanistic work from Kariyawasam et al., who showed that human resistin-dependent NLRP3 inflammasome activation in macrophages promoted IL-1β and IL-18 secretion and enhanced human pulmonary vascular smooth muscle cell proliferation [67]. Together, these studies strengthen the evidence linking human resistin to inflammatory pulmonary vascular remodeling and disease severity, although neither study establishes a direct role in parenchymal pulmonary fibrosis.

Neumann et al. found that adiponectin is highly expressed in healthy lungs but markedly reduced as fibrosis progresses [114]. In contrast, overall resistin expression was present across all lung tissue areas with no relevant difference between fibrotic and control samples; however, resistin-positive cells were characteristically increased within the immune cell infiltrates of idiopathic pulmonary fibrosis (IPF) and systemic sclerosis (SSc) tissues [114].

Liu et al. also showed that *FIZZ2* is nearly absent in healthy lungs but is strongly induced early after bleomycin-induced injury [36]. *FIZZ2*-knockout mice exhibited significantly reduced fibrosis despite persistent inflammation, demonstrating that *FIZZ2* is required for fibrotic progression. Mechanistically, *FIZZ2* activates lung fibroblasts by promoting fibroblast proliferation, type I collagen production, and myofibroblast differentiation through ERK/MAPK signaling [36]. Another study similarly reported that RELMβ expression is markedly increased under hypoxic conditions—particularly in lung epithelial cells—and is also induced in pulmonary smooth muscle cells and fibroblasts. Overexpression of *RELMβ* enhanced epithelial and smooth muscle cell proliferation via PI3K signaling [35].

In cystic fibrosis, resistin accumulates within the lung, with sputum concentrations 50–100-fold higher than plasma levels; resistin was undetectable in sputum from healthy individuals. Increasing sputum resistin levels correlated with worsening lung function, suggesting that resistin reflects airway inflammation rather than structural fibrotic remodeling in cystic fibrosis [45].

Human resistin has also been linked to dermatomyositis-associated interstitial lung disease (DM-ILD) [4]. In this setting, resistin mRNA levels in peripheral blood mononuclear cells (PBMCs) were increased in patients with DM-ILD and were particularly elevated in rapidly progressive interstitial lung disease (RP-ILD) [4]. Resistin expression was also detected in DM-ILD lung tissue, mainly in macrophages and alveolar epithelial cells, with weaker staining in fibrotic lesions and they further showed that PBMC resistin mRNA levels negatively correlated with diffusing capacity for carbon monoxide (DLCO) and positively correlated with inflammatory and disease activity markers [4]. Similarly, Angelini et al. reported that RELMβ is markedly upregulated in the lungs of patients with scleroderma-associated pulmonary hypertension and promotes endothelial and smooth muscle cell proliferation via ERK1/2 signaling [115]. Extending these observations, Liu et al. reported that RELMβ directly interacts with the calcium-sensing receptor (CaSR) and regulates PLC–IP_3_R-dependent intracellular Ca^2+^ signaling in hypoxia-induced pulmonary hypertension, thereby promoting pulmonary artery smooth muscle cell proliferation [116]. This study further defines a RELMβ-specific mechanism of pulmonary vascular remodeling but does not provide direct evidence for human resistin or parenchymal pulmonary fibrosis. [116].

Yamaji-Kegan et al. showed that hypoxia induces murine RELMα (HIMF) expression independently of T helper 2 (Th2) cytokines [42]. However, the pathological consequences of HIMF—including pulmonary vascular cell proliferation, extracellular matrix and collagen accumulation, macrophage recruitment, and endothelial activation—were dependent on interleukin-4 (IL-4)/IL-4 receptor α (IL-4Rα) signaling [42]. RELMα also enhanced production of vascular endothelial growth factor (VEGF), MCP-1, and stromal cell–derived factor-1 (SDF-1), contributing to a pro-angiogenic and chemotactic lung microenvironment [42].

Mishra et al. investigated murine RELMβ in allergen-driven Th2 lung inflammation and remodeling [117]. Using ovalbumin- and Aspergillus fumigatus–induced asthma models, they found that RELMβ is strongly induced through IL-4/Interleukin-13(IL-13)–signal transducer and activator of transcription 6 (STAT6) signaling and is predominantly expressed in airway epithelium and peribronchial and perivascular regions [117]. Intratracheal administration of recombinant RELMβ induced marked collagen deposition, whereas genetic deletion of *RELMβ* significantly reduced collagen accumulation and goblet cell hyperplasia without altering inflammatory cell infiltration [117].

Although several studies indicate that resistin-like molecules can promote fibrotic remodeling, not all RELM proteins are sufficient to induce fibrosis independently. Madala et al. demonstrated that epithelial overexpression of murine RELMα increased dendritic cell accumulation in the lung but did not induce collagen deposition, myofibroblast expansion, or structural remodeling, even after prolonged expression or in bleomycin- and silica-induced injury models [118]. Pesce et al. further showed that RELMα functions as a protective immunoregulatory molecule rather than a profibrotic factor. In a helminth-induced Th2 inflammation model, RELMα deficiency resulted in exaggerated IL-4/IL-13 responses accompanied by increased lung and liver inflammation and fibrosis [119]. The pulmonary evidence should be interpreted with particular caution. Human resistin has been associated with inflammatory cell infiltration, pulmonary vascular remodeling, and disease activity in selected lung disorders [4,104,105], but direct evidence that human resistin itself drives pulmonary fibrosis remains limited. Most mechanistic studies demonstrating fibroblast activation, myofibroblast differentiation, or collagen deposition involve rodent *RELMα/FIZZ1* or *RELMβ/FIZZ2* rather than human resistin [35,36,40,42,117,118,119]. These findings support an important role for the broader RELM/FIZZ family in pulmonary remodeling, but they should not be considered direct proof that human resistin exerts identical profibrotic effects.

### 6.4. Renal Disease: Associative Evidence

In the kidney—where chronic inflammation and sustained interactions between immune cells and resident renal cells drive extracellular matrix accumulation—members of the resistin family have been investigated in renal disease and fibrosis-related outcomes. However, the literature has largely centered on chronic kidney disease (CKD) and related renal disorders, with relatively few studies directly addressing fibrotic remodeling.

In a cohort of 239 CKD patients, Axelsson et al. demonstrated that circulating resistin levels are significantly elevated in CKD and increase progressively with declining glomerular filtration rate (GFR) [120]. Serum resistin concentrations showed an inverse correlation with GFR and a positive association with systemic inflammatory markers, including C-reactive protein (CRP), IL-6, and TNF-α. Multivariate analyses indicated that reduced renal function was the primary determinant of elevated resistin levels, whereas associations with insulin resistance and adiposity diminished after adjustment for GFR [120].

Romejko et al. similarly reported that resistin is associated with increased cardiovascular risk in non-dialyzed male CKD patients [121]. Circulating resistin levels were higher than in healthy controls and rose progressively with decreasing estimated GFR (eGFR), with the highest levels observed in individuals at elevated cardiovascular risk [121]. Resistin concentrations correlated positively with plasminogen activator inhibitor-1 (PAI-1), a marker of thrombotic risk, and with TNF-α, but showed no association with body mass index (BMI) [121].

Risch et al. examined adipokine profiles in patients with coronary artery disease and normal or mildly impaired renal function [122]. They found that serum resistin levels increased as GFR declined, and this inverse association remained significant after adjustment for age, BMI, diabetes, lipid profile, and other cardiovascular risk factors. Notably, the relationship was present only when renal function was reduced; no significant association was observed in individuals with normal GFR [122].

Tsioufis et al. evaluated circulating resistin levels in 132 newly diagnosed, untreated hypertensive individuals with preserved renal function to determine whether resistin is associated with early kidney injury independent of diabetes or antihypertensive therapy [123]. Resistin levels were independently associated with increased urinary albumin excretion after adjustment for eGFR, blood pressure, and metabolic factors, suggesting a link between resistin and early microvascular or endothelial injury rather than impaired renal clearance [123].

Ellington et al. assessed whether circulating resistin reflects early kidney injury in hypertensive adults before overt renal dysfunction [124]. In a large cross-sectional cohort of 1575 hypertensive individuals without established cardiovascular disease, higher plasma resistin levels were independently associated with lower eGFR after adjustment for metabolic and inflammatory variables, indicating early impairment of renal clearance rather than nonspecific metabolic effects [124]. Resistin was also associated with albuminuria, but only in hypertensive patients with diabetes, supporting a link to glomerular microvascular stress under metabolic strain [124].

Díez et al. investigated determinants of elevated resistin levels in patients with end-stage renal disease (ESRD). Resistin concentrations were markedly increased across patients receiving hemodialysis, peritoneal dialysis, or conservative management, independent of BMI, insulin resistance, and other metabolic factors. Associations with cardiovascular disease were limited to a history of heart disease, with no significant relationship observed for cerebrovascular or peripheral vascular disease [125]. However, these observations may partly reflect impaired renal clearance and systemic inflammatory burden, and direct evidence that human resistin activates renal fibroblasts or independently promotes extracellular matrix deposition remains limited. Resistin should therefore currently be viewed primarily as a disease-associated and inflammatory biomarker in renal disorders, with its direct contribution to renal fibrogenesis requiring further experimental investigation. Current renal evidence is predominantly associative rather than mechanistic. Circulating resistin consistently increases as renal function declines and is associated with inflammation, albuminuria, and cardiovascular risk in CKD populations [120,121,122,123,124,125]. Resistin should therefore currently be viewed primarily as a disease-associated and inflammatory biomarker in renal disorders, with its direct contribution to renal fibrogenesis requiring further experimental investigation.

Taken together, the available evidence differs substantially across organ systems in terms of the molecules examined, experimental approach, mechanistic directness, and clinical relevance. To distinguish direct mechanistic findings from human tissue observations and circulating biomarker associations, the relative strength and principal limitations of the evidence are summarized in Table 2.

## 7. A Unifying Hypothesis: The “Fibrotic Switch”

The evidence across organ systems supports a unified hypothesis: Persistent inflammation, as seen in metabolic syndrome, leads to chronically elevated resistin, which acts as a “fibrotic switch.” This constant signal locks fibrogenic cells into a perpetually activated, proliferative state, shifting the ECM balance from degradation toward excessive synthesis, leading to collagen deposition and tissue stiffening. The resulting fibrosis impairs organ function, which in turn exacerbates metabolic disturbances and inflammation, further fueling resistin production and creating a vicious cycle that drives disease progression (Table 3).

The evidence across organ systems supports a unifying mechanistic hypothesis in which chronically elevated resistin functions as a “fibrotic switch” by coupling persistent inflammatory and metabolic stress to fibrogenic remodeling. In this framework, resistin does not replace inflammation as its established pathogenic context; rather, it extends inflammatory signaling into a tissue-remodeling program. Through receptor- and cell-context-dependent pathways, resistin may amplify inflammatory and cellular stress responses while also favoring profibrotic cellular programs, increased extracellular matrix synthesis, and impaired matrix resolution. The balance may consequently shift from adaptive tissue repair toward pathological collagen accumulation, tissue stiffening, and progressive structural remodeling.

The operation of this proposed switch is likely to vary according to species, cellular source, receptor availability, target-cell phenotype, and organ context. Direct profibrotic effects are most clearly supported in selected cardiac and hepatic experimental systems, whereas evidence in pulmonary and renal disease is more heterogeneous and, in the lung, frequently derives from other RELM/FIZZ family members. Accordingly, the “fibrotic switch” is presented here as a unifying conceptual framework grounded in the currently available mechanistic evidence, rather than as a single pathway already established across all tissues. Further longitudinal and mechanistic studies are needed to define its organ-specific validity, causal boundaries, and translational relevance. Nevertheless, once fibrotic remodeling compromises organ function, the resulting metabolic and inflammatory stress may further increase resistin production, creating a vicious cycle that promotes continued tissue remodeling and disease progression (Table 3).

## 8. Therapeutic Implications and Future Directions

Positioning resistin as a mechanistic link between inflammatory signaling and fibrogenic remodeling opens potential therapeutic avenues. Rather than targeting only downstream consequences such as extracellular matrix accumulation, interventions could also disrupt resistin-dependent pathways involved in the initiation or amplification of fibrogenic responses. Potential strategies include: (1) developing monoclonal antibodies or other agents that sequester circulating resistin; (2) identifying antagonists of the candidate receptors or binding partners involved in context-dependent profibrotic signaling; (3) inhibiting intracellular pathways activated by resistin in fibrogenic cells; and (4) targeting transcriptional mechanisms that regulate resistin expression. For example, we previously showed that genetic or pharmacological restoration of SERCA2a function using the small-molecule SERCA2 activator CDN1163 suppressed resistin expression through inhibition of NFATc signaling in diabetic hearts [126].

Each of these strategies presents important translational challenges. Direct neutralization of circulating resistin may offer greater ligand specificity than inhibition of downstream pathways, but its efficacy could vary if locally produced or tissue-associated resistin contributes substantially to disease. Experimental proof of concept has recently emerged in pulmonary vascular models: a human resistin-blocking monoclonal antibody attenuated NLRP3 inflammasome activation and downstream pulmonary vascular smooth muscle cell proliferation [67]. However, this study evaluated inflammatory pulmonary vascular remodeling rather than parenchymal or organ fibrosis. Moreover, because resistin has antimicrobial and immunomodulatory functions, prolonged systemic neutralization may interfere with host-defense responses [33]. Receptor-directed approaches also require caution. CAP1 mediates human resistin signaling in monocytes [53], but it also has established roles in cofilin regulation, actin cytoskeletal dynamics, and cell adhesion [127,128,129]. Consequently, global CAP1 inhibition could disrupt cellular functions beyond resistin signaling and has not yet been established as an antifibrotic strategy. TLR4 inhibition has shown antifibrotic activity in several preclinical models, including models relevant to systemic sclerosis, but TLR4 is a broadly acting innate immune receptor rather than a resistin-specific target [130]. Consequently, systemic TLR4 blockade could affect antimicrobial sensing and signaling initiated by multiple endogenous and microbial ligands. These considerations favor tissue-targeted approaches or agents that selectively disrupt resistin–receptor interactions rather than globally suppressing CAP1 or TLR4 function.

Future research should focus on defining the receptor or binding-partner interactions that mediate resistin-associated fibrogenic responses; using cell-specific knockout models to distinguish the contributions of immune-cell-derived and locally produced resistin to organ fibrosis; determining whether longitudinal changes in circulating or tissue-associated resistin are associated with fibrosis progression or regression; and testing whether anti-resistin strategies can attenuate fibrotic remodeling in preclinical models of MASH, heart failure, and other fibrotic diseases. Within the studies reviewed here, direct anti-resistin neutralization has not been established as an antifibrotic therapy, and the clinical literature has largely evaluated resistin as a biomarker rather than as a therapeutic target. Carefully designed preclinical studies will therefore be required to establish efficacy, optimal tissue exposure, safety, and potential effects on immune defense before clinical translation can be considered.

## 9. Conclusions

Taken together, the available evidence supports a paradigm shift that expands resistin biology beyond its established roles in insulin resistance and inflammation. Rather than replacing these established functions, the framework proposed here identifies an additional mechanistic role in which resistin may couple chronic inflammatory and metabolic stress to stromal cell activation and extracellular matrix remodeling. In selected tissue contexts, resistin can act directly on fibrogenic cells and may thereby help initiate or sustain structural organ damage. Resistin may therefore function as a context-dependent “fibrotic molecular switch” linking persistent inflammatory signaling to progressive tissue remodeling. However, this framework remains a unifying hypothesis rather than a universally established mechanism and requires further organ-, species-, and cell-specific validation. Further definition of the receptors, cellular sources, and organ-specific mechanisms underlying this axis may reveal new opportunities for biomarker development and therapeutic intervention.

## Figures and Tables

**Table 1:** Comparison of molecular, genetic, and cellular characteristics of mouse and human resistin.

Feature Category	Mouse Resistin	Human Resistin	References
**Molecular weight**	11 kDa	12.5 kDa	[3,33,46]
***RETN*** **gene localization**	Chromosome 8a1	Chromosome 19p13.2	[11,33]
**Protein processing and structure**	Secreted as a polypeptide precursor and undergoes post-translational cleavage	Secreted protein primarily composed of an alpha-helical coiled-coil domain	[3,27,33,43,44]
**Oligomerization pattern**	Forms disulfide bond–dependent oligomers, including trimers (most active form) and hexamers	Oligomerization depends on a critical cysteine residue at position 6	[33,43,44]
**Primary cellular source**	Predominantly expressed in white adipose tissue	Predominantly expressed in immune cells, including macrophages, monocytes, and neutrophils	[10,33,37,38,39]
**Distinct structural features**	Lacks RGD domain	Contains an RGD domain absent in mouse resistin	[27,46]

**Table 2:** Evidence strength reflects the directness, consistency, and translational relevance of the available findings rather than a formal systematic quality-grading procedure. “Strong” indicates multiple experimental perturbation studies with fibrotic or remodeling outcomes; “moderate” indicates supportive but incomplete or heterogeneous mechanistic or human evidence; and “limited” indicates predominantly associative, indirect, or family-member-based evidence. Findings involving RELM/FIZZ proteins were not considered direct evidence of human resistin activity.

Organ	Molecule(s) Predominantly Evaluated	Experimental/ Mechanistic Evidence	Human Tissue/ Translational Evidence	Clinical/ Biomarker Evidence	Overall Interpretation
**Heart**	Murine and human resistin; *Retnlα* in ischemic injury	**Strong.** Genetic deletion, cardiac overexpression, and cell-based studies support direct effects on fibroblast-to-myofibroblast differentiation, profibrotic signaling, apoptosis, and adverse cardiac remodeling [79,81,82,84,85,92].	**Moderate.** Human endothelial-cell studies support vascular and oxidative-stress effects, while human resistin has also shown membrane-disruptive activity in experimental ischemic settings [68,89].	**Moderate but** **associative.** Circulating resistin is associated with myocardial injury, heart failure, and cardiovascular events, although an association with myocardial fibrosis has not been consistently demonstrated. [17,88,93]	Cardiac literature provides the strongest evidence for a direct profibrotic role, but the specificity of circulating resistin as a marker of myocardial fibrosis remains uncertain.
**Liver**	Human and rodent resistin	**Moderate.** Experimental studies demonstrate effects on hepatic stellate-cell behavior, Kupffer-cell signaling, inflammatory mediator production, and profibrotic gene expression [60,80,100].	**Moderate.** Human liver studies show localization of resistin within inflammatory and fibrogenic regions and associations between intrahepatic resistin expression and fibrosis severity [60,97,101,104].	**Heterogeneous.** Some studies associate circulating resistin with advanced fibrosis or cirrhosis, whereas others report no independent relationship with histological severity [98,102,105,106,107,108,109].	Hepatic evidence supports context-dependent profibrotic activity, but circulating resistin is not a consistent fibrosis biomarker across patient populations.
**Lung**	Human resistin; predominantly *RELMα/FIZZ1* and *FIZZ2/FIZZ2* in mechanistic studies	**Moderate** at the RELM/FIZZ- family level but limited for human *RETN* itself. *RELMα/FIZZ1* and *FIZZ2/FIZZ2* can promote fibroblast activation, myofibroblast differentiation, collagen deposition, and pulmonary remodeling [35,36,40,42,117,118,119]. Human resistin has direct mechanistic support in macrophage-driven NLRP3 inflammasome activation and pulmonary vascular smooth muscle cell proliferation, but not in parenchymal pulmonary fibrosis [67,112]	**Limited to moderate.** Human resistin has been detected in inflammatory cells and associated with pulmonary vascular remodeling and disease activity, but direct human- resistin-mediated fibrosis has not been established [4,112,114,115].	**Limited.** Available clinical observations primarily associate resistin with inflammation, lung-function impairment, or disease activity rather than direct quantification of fibrotic progression [4,45,114].	Pulmonary data support an important role for the wider RELM/FIZZ family, but findings involving these proteins should not be considered direct evidence for identical profibrotic actions of human *RETN*.
**Kidney**	Predominantly circulating human RESISTIN.	**Limited.** Direct evidence that resistin independently activates renal fibroblasts or promotes renal extracellular matrix deposition is currently lacking in cited literature.	**Limited.** Available studies suggest associations with endothelial or microvascular injury but no direct tissue-level evidence of renal fibrogenesis	**Moderate but associative.** Circulating resistin consistently correlates with declining renal function, systemic inflammation, albuminuria, and cardiovascular risk in CKD populations [120,121,122,123,124,125].	Renal evidence is predominantly biomarker-based and may partly reflect impaired clearance and systemic inflammation; a direct causal role in renal fibrosis remains unproven.

**Table 3:** Organ-specific evidence linking human and rodent resistin and individual RELM/FIZZ family members to tissue remodeling and fibrosis.

Organ	Study Model	Molecule Evaluated	Change or Exposure	Principal Finding	Mechanism	Ref.
**Heart**	AAV9-mediated cardiac overexpression of mouse *Retn* in normal rats	Murine resistin (mouse *Retn* transgene)	Cardiac *Retn* overexpression	Systolic dysfunction and cardiac remodeling, leading to myocardial fibrosis, apoptosis, and Ca^2+^ dyshomeostasis	Oxidative stress via NADPH oxidase; TNF-α/NF-κB signaling; decreased SERCA2a/Phospholamban ratio	[81]
**Heart**	Sprague–Dawley rats and rat A10 vascular smooth muscle cells	Recombinant mouse resistin	Resistin exposure before endothelin-1 stimulation	Strengthens and prolongs endothelin-1 vasoconstrictor effect	SOCE activation	[83]
**Heart**	Adipose tissue-specific Retn-knockout mice and AAV9-mediated cardiac *Retn* overexpression under transverse aortic constriction	Murine resistin (*Retn*)	*Retn* deletion versus cardiac *Retn* overexpression	Deletion attenuates pressure overload cardiac fibrosis with decreased fibrotic markers; overexpression shows the opposite.	Gadd45a/miR-148b-3p; DNA damage response; cardiomyocyte apoptosis and fibrosis	[82]
**Heart**	H9C2 cardiomyoblast	Recombinant human RETN	Resistin exposure	Induces hypertrophy with increased BNP and β-MHC expression	Hypertropic gene expression	[84]
**Heart**	H9C2 cardiomyoblast	Recombinant human RETN and recombinant human omentin-1	Resistin exposure with or without omentin-1 treatment	Omentin attenuates resistin-induced hypertrophy on H9C2 cells	Antagonizing TLR4/MyD88/NF-κB/ERK pathway	[85]
**Heart**	NIH-3T3 fibroblasts, adult mouse cardiac fibroblasts, and HFD-challenged *Retn*-knockout mice	Recombinant mouse Retn and endogenous murine Retn	Recombinant RETN exposure and genetic *Retn* deletion	Resistin induced fibroblast-to-myofibroblast differentiation and increased *α-SMA*, *Col1a1*, *fibronectin*, *Ccn2*, *and Mmp9*; *Retn* deletion reduced cardiac fibrosis	JAK2/STAT3 and JNK/c-Jun activation; independent of Smad3/TGF-β pathway	[79]
**Heart**	MESA cohort of adults without baseline cardiovascular disease	Circulating human RETN	Higher plasma resistin levels	Increased HFrEF risk but no relationship with myocardial fibrosis	No direct association with myocardial fibrosis was observed	[17]
**Heart**	Ischemia– reperfusion	Circulating human RETN	Perioperative and reperfusion-associated increase in plasma resistin	Associated with myocardial injury and oxidative stress; suggested biomarker potential	Associated with myocardial injury- related troponin T and oxidative stress	[88]
**Heart**	Human coronary artery endothelial cells	Recombinant human RETN	resistin exposure	Decreased eNOS mRNA and protein and intracellular NO, indicating endothelial dysfunction	ROS generation and p38/JNK activation; reduced *eNOS* mRNA stability and NO bioavailability	[68]
**Heart**	Rat pressure- and volume-overload hypertrophy models, chronic myocardial infarction, and cardiomyocyte studies	Rat resistin	Altered myocardial resistin expression and resistin exposure	Pressure overload hypertrophy characterized by fibrosis; resistin elevated in pressure overload and chronic ischemic injury; linked to ischemia mediated cardiac fibrosis	Promoted profibrotic signaling through *CTGF*	[92]
**Heart**	MESA cohort of adults without baseline cardiovascular disease	Circulating human resistin	Higher circulating resistin	Higher incidence of HF, CHD, and overall CVD	Heart failure and coronary heart disease	[93]
**Heart**	TAC/DOCA- induced HFpEF mouse model	Murine RELMγ and ADCY1	Induction of HFpEF by transverse aortic constriction and deoxycorticosterone acetate treatment	Myocardial fibrosis was accompanied by increased Relmγ and reduced Adcy1 expression	Transcriptomic and expression analyses identified a RELMγ–ADCY1 axis associated with HFpEF-related fibrosis; direct causality was not established	[90]
**Liver**	Patients with chronic hepatitis B receiving antiviral therapy	Resistin	Higher serum resistin	Higher resistin in more advanced fibrosis; adiponectin not significantly associated	Association with fibrosis severity	[97]
**Liver**	Chronic liver injury/end-stage liver disease/acute alcoholic hepatitis; primary human HSC cell line	Circulating human resistin	Resistin upregulated in chronic injury; recombinant resistin exposure	Localizes to inflammation/fibrogenesis areas; induces MCP-1 and IL-8; enhances monocyte chemotaxis; does not promote HSC proliferation or collagen I/TGF-β1 expression	NF-κB–dependent MCP-1 and IL-8 induction; no collagen I/TGF-β1 induction	[60]
**Liver**	Cross-sectional study of patients with NAFLD and healthy controls	Circulating human RETN	Higher serum resistin in NAFLD vs. controls	No difference between mild vs. advanced fibrosis; no association with fibrosis severity	No association with fibrosis severity reported	[98]
**Liver**	High-fat diet NAFLD model and in vitro HSC	Rat resistin	Time-dependent hepatic resistin increase; recombinant resistin exposure	Progressive fibrosis with increased serum fibrosis markers; recombinant resistin increases fibrotic marker release and upregulates TGF-β1 and TNF-α	TGF-β1 and TNF-α upregulation; fibrotic marker increase	[100]
**Liver**	Pediatric NAFLD patients	Tissue- associated human resistin	Resistin-positive hepatic progenitor cells (HPC) increased	Resistin-positive HPC number correlates with fibrosis severity	Correlation between resistin-positive HPCs and fibrosis severity	[101]
**Liver**	Liver cirrhosis patients	Circulating human resistin	Higher fasting plasma resistin	Higher resistin parallel with severity of liver dysfunction	Parallel with disease severity	[102]
**Liver**	Bile duct ligation in Sprague– Dawley rats and primary rat hepatic stellate cell and Kupffer cell studies	Rat resistin	Circulating resistin is increased; hepatic resistin is unchanged; recombinant resistin exposure	Resistin promotes HSC proliferation/migration and inhibits apoptosis; activates Kupffer cells to increase TGF-β1; factors from resistin-treated Kupffer cells promote collagen I and CTGF in HSC; described as modulator	p38 MAPK activation; IL-6- and MCP-1- dependent HSC responses; Kupffer cell-derived TGF-β1 mediated indirect collagen I and CTGF induction	[80]
**Liver**	MASH patients	Circulating and tissue- associated human RETN	Increased hepatic resistin mRNA and protein expression in MASH	Circulating RETN elevated in NAFLD but not distinguishing MASH vs. steatosis; hepatic resistin associated with lobular inflammation and fibrosis stage; localized to perisinusoidal cells, HSC, Kupffer cells	Hepatic resistin expression and cellular localization correlates with inflammatory and fibrotic activity	[104]
**Liver**	NAFLD cohort study	Circulating human RETN	Higher serum RETN in advanced fibrosis	Suggested as potential serum biomarker for advanced hepatic fibrosis identification	Biomarker suggestion	[105]
**Liver**	Cross-sectional study of patients with MASH or simple steatosis and obese and non-obese controls	Circulating human RETN	No significant differences across MASH/ steatosis/obese controls	Not independently associated with severity; did not predict fibrosis presence or stage	No independent association reported	[106]
**Liver**	Obese children with and without NAFLD and non-obese controls	Circulating human RETN	No differences across groups	Not associated with hepatic steatosis presence and severity, insulin resistance, or liver injury markers	No association reported	[107]
**Liver**	NAFLD case– control	Circulating human RETN	No consistent difference between controls vs. patients	Not consistently associated with steatosis grade; higher in moderate-to-severe fibrosis vs. mild.	Mixed/limited association described	[108]
**Liver**	Patients with liver cirrhosis and healthy controls	Circulating human RETN	Elevated resistin levels; increases with disease stage	Suggests active hepatic resistin production; linked to proinflam- matory state; no association with insulin resistance measures	Correlation with TNF-α; no insulin resistance association	[109]
**Lung**	Bleomycin- induced rat model	Rat RELMα/ FIZZ1	significantly upregulated; expressed in alveolar type II and airway epithelial cells	Epithelial-derived RELMα promotes fibroblast-to-myofibroblast differentiation with increased α-SMA and type I collagen; no involvement of TGF-β signaling	Direct effect independent of TGF-β signaling	[40]
**Lung**	Human idiopathic pulmonary hypertension lung tissue, hypoxia- induced pulmonary hypertension in mice, and macrophage– pulmonary artery smooth muscle cell experiments	Human RETN and murine RELMα/ FIZZ1 evaluated separately within the same study	Increased human resistin expression in human lung tissue; hypoxia-induced *RELMα/FIZZ1* expression and recombinant protein exposure in experimental models	Human RETN was increased in macrophage-like inflammatory cells in pulmonary hypertension, whereas murine RELMα/FIZZ1 promoted macrophage accumulation and macrophage-dependent pulmonary artery smooth muscle cell proliferation, supporting pulmonary vascular remodeling	HMGB1/RAGE-dependent smooth muscle cell proliferation, BTK-mediated macrophage migration, and RELMα-associated suppression of macrophage Sirt1 signaling	[112]
**Lung**	Human systemic sclerosis, idiopathic pulmonary fibrosis, and control lung tissues	Tissue- associated human RETN	Resistin rarely detected in fibrotic tissue/collagen-rich areas/fibroblasts; localized to inflammatory immune-cell regions	Suggests association with inflammation rather than direct fibrotic areas	Localization of resistin is reported	[114]
**Lung**	Bleomycin- induced pulmonary fibrosis in wild-type and *Retnlβ*-deficient mice, with lung fibroblast studies	Murine RELMβ/ FIZZ2	Strongly induced early after injury; absent in healthy lungs	*FIZZ2*-knockout shows reduced fibrotic response despite persistent inflammation	Activates fibroblasts via ERK/MAPK; promotes proliferation, collagen I, myofibroblast differentiation	[36]
**Lung**	Human lung epithelial cells, pulmonary artery smooth muscle cells, and fibroblast-derived cells under hypoxia	Human RELMβ/ FIZZ2	Increased under hypoxia; induced in smooth muscle cells and fibroblasts	Overexpression enhances proliferation in epithelial and smooth muscle cells	PI3K-dependent mitogenic signaling; vascular/remodeling evidence rather than direct fibrosis evidence	[35]
**Lung**	Cystic fibrosis patients	Sputum and circulating human RETN	Accumulates in lung; sputum 50–100× higher than plasma; significantly lower in healthy sputum	Increasing sputum resistin associated with worsening lung function; reflects airway inflammation rather than structural fibrotic remodeling	Biomarker of neutrophil-dominant airway inflammation rather than direct evidence of fibrotic remodeling	[45]
**Lung**	Patients with dermatomyositis- associated interstitial lung disease, including lung tissue and PBMC analyses	Human RETN	Present in fibrotic lung tissue; higher in rapidly progressive ILD	Negative correlation with DLCO; persistent inflammation with resistin paralleling inflammatory markers; decreases after immunosuppression	Parallel changes with inflammatory markers; decreases after treatment	[4]
**Lung**	Human scleroderma-associated pulmonary hypertension lung tissue and cultured pulmonary endothelial and smooth muscle cells	Human RELMβ/ FIZZ2	Significantly upregulated	Promotes pulmonary endothelial and smooth muscle cell proliferation	ERK1/2-dependent mitogenic signaling; evidence of pulmonary vascular remodeling rather than direct parenchymal fibrosis	[115]
**Lung**	Hypoxia-induced pulmonary hypertension in mice	Murine RELMα/ FIZZ1	Hypoxia induces HIMF independently of Th2 cytokines	Pathological consequences such as vascular proliferation, ECM/ collagen accumulation, macrophage recruitment, endothelial activation observed dependent on IL-4/IL-4Rα	IL-4/IL-4Rα -dependence; increased VEGF, MCP-1, SDF-1	[42]
**Lung**	OVA- and Aspergillus-induced murine allergic airway models, recombinant protein administration, and Retnlβ deletion	Murine RELMβ/ FIZZ2	Allergen-induced expression, recombinant RELMβ administration, and genetic deletion	Recombinant RELMβ leads to collagen deposition; deletion reduces collagen accumulation and goblet cell hyperplasia without altering inflammatory infiltration	IL-4/IL-13–STAT6- dependent induction with direct remodeling and fibroblast- motogenic activity	[117]
**Lung**	Lung epithelial *RELMα/FIZZ1* overexpression with pulmonary injury models in mice	Murine RELMα/ FIZZ1	Epithelial overexpression	Increased dendritic cells but no collagen deposition, myofibroblast accumulation, or structural remodeling	*RELMα/FIZZ1* expression alone was insufficient to induce pulmonary fibrosis in this model	[118]
**Lung**	Helminth-induced Th2 inflammation in *Retnlα*- deficient mice	Murine RELMα/FIZZ1	Genetic *Retnlα* deletion	Exaggerated IL-4/IL-13 responses with increased pulmonary granulomatous inflammation and augmented hepatic inflammation and fibrosis	Protective and immunoregulatory role described	[119]
**Lung**	Bleomycin- induced pulmonary fibrosis in rats	Murine RELMα/ FIZZ1	Bleomycin exposure with or without trimetazidine treatment	Trimetazidine reduced FIZZ1 expression, hydroxyproline accumulation, and histological and molecular indices of pulmonary fibrosis	Modulation of the lncRNA CBR3-AS1/miR-29/*FIZZ1* axis, with reduced TGF-β1 and Smad3 signaling; *FIZZ1* was not selectively manipulated	[41]
**Lung**	Human macrophages and human pulmonary vascular smooth muscle cells	Human RETN	Recombinant human resistin exposure and resistin pathway inhibition	Resistin promoted IL-1β and IL-18 release and enhanced pulmonary vascular smooth muscle cell proliferation	HMGB1-dependent NF-κB priming and BTK-dependent NLRP3 phosphorylation and inflammasome activation	[67]
**Lung**	Clinical cohort of 1121 adults with pulmonary arterial hypertension	Circulating human RETN	Measurement of serum resistin concentrations	Higher resistin levels were associated with shorter six-minute walking distance, reduced cardiac index, and increased mortality risk	Mechanism was not directly assessed. Findings provide associative clinical/ biomarker evidence	[113]
**Lung**	Hypoxia-induced pulmonary hypertension model and pulmonary artery smooth muscle cells	RELMβ/ FIZZ2	Hypoxic exposure with RELMβ pathway evaluation	RELMβ promoted intracellular Ca^2+^ signaling and pulmonary artery smooth muscle cell proliferation	Direct interaction with CaSR and activation of the PLC–IP3R- dependent Ca^2+^ signaling pathway	[116]
**Kidney**	Patients with chronic kidney disease	Circulating human RETN	Elevated resistin; increases with reduced GFR	Inverse correlation with GFR; positive association with CRP, IL-6, TNF-α; multivariate: reduced renal function accounts for elevation	Associations with inflammatory markers; attenuation after GFR adjustment	[120]
**Kidney**	Non-dialyzed male CKD patients	Circulating human resistin	Elevated RETN; increases with reduced eGFR	Higher in high cardiovascular risk; associated with PAI-1 and TNF-α; no association with BMI	Association with thrombotic risk marker and TNF-α	[121]
**Kidney**	Coronary artery disease with normal and mildly impaired kidney function	Circulating human RETN	Increases as GFR declines	Inverse association remains after multiple adjustments; present only when renal function reduced	Renal function dependent association	[122]
**Kidney**	Newly diagnosed untreated hypertensive individuals with preserved GFR	Circulating human RETN	Circulating levels of RETN	Independently associated with increased urinary albumin excretion after adjustments	Link suggested to early microvascular and endothelial kidney injury rather than clearance	[123]
**Kidney**	Hypertensive cohort study with hypertension patients	Circulating human RETN	Higher plasma RETN	Independently associated with lower eGFR after adjustments; associated with albuminuria only in diabetics	Early impairment of renal clearance; glomerular microvascular stress under metabolic stress	[124]
**Kidney**	Patients with end-stage renal disease receiving hemodialysis, peritoneal dialysis, or conservative treatment	Circulating human RETN	Significantly increased	Independent of BMI, insulin resistance, metabolic factors; CVD association limited to history of heart disease	Determinants/ associations described	[125]

## Data Availability

No new data were created or analyzed in this study. Data sharing is not applicable to this article.

## Abbreviations

ADCY1	Adenylyl cyclase 1
AKT	Protein kinase B
ALT	Alanine aminotransferase
AMPK	Adenosine monophosphate-activated protein kinase
ANF	Atrial natriuretic factor
BMI	Body mass index
BNP	Brain natriuretic peptide
BTK	Bruton’s tyrosine kinase
cAMP	Cyclic adenosine monophosphate
CAP1	Adenylyl cyclase-associated protein 1
Ca^2+^	Calcium ion
Cav1.2	L-type voltage-gated calcium channel Cav1.2
CB1R	Cannabinoid receptor type 1
CCN2	Cellular communication network factor 2
CHD	Coronary heart disease
CKD	Chronic kidney disease
c-Jun	Jun proto-oncogene/AP-1 transcription factor subunit
Col1a1	Collagen type I alpha 1
CPT1A	Carnitine palmitoyltransferase 1A
CREB	cAMP response element-binding protein
CRP	C-reactive protein
CSF	Cerebrospinal fluid
CaSR	Calcium-sensing receptor
CTGF	Connective tissue growth factor
CVD	Cardiovascular disease
DLCO	Diffusing capacity for carbon monoxide
DM-ILD	Dermatomyositis-associated interstitial lung disease
DNA	Deoxyribonucleic acid
DOCA	Deoxycorticosterone acetate
ECM	Extracellular matrix
eGFR	Estimated glomerular filtration rate
eNOS	Endothelial nitric oxide synthase
ER	Endoplasmic reticulum
ERK	Extracellular signal-regulated kinase
ERK1/2	Extracellular signal-regulated kinase 1/2
ESRD	End-stage renal disease
FIZZ	Found in inflammatory zone
*FIZZ1*	Found in inflammatory zone 1
*FIZZ2*	Found in inflammatory zone 2
*FIZZ3*	Found in inflammatory zone 3
Gadd45a	Growth arrest and DNA damage-inducible alpha
GFR	Glomerular filtration rate
GGT	γ-glutamyl transpeptidase
GLUT1	Glucose transporter type 1
GLUT4	Glucose transporter type 4
HF	Heart failure
HFrEF	Heart failure with reduced ejection fraction
HIMF	Hypoxia-induced mitogenic factor
HMGB1	High mobility group box 1
HPC	Hepatic progenitor cell
HSC	Hepatic stellate cell
HSC-T6	HSC-T6 hepatic stellate cell line
I/R	Ischemia–reperfusion
IGF-1R	Insulin-like growth factor 1 receptor
IL-1β	Interleukin-1 beta
IL-4	Interleukin-4
IL-4Rα	Interleukin-4 receptor alpha
IL-6	Interleukin-6
IL-8	Interleukin-8
IL-13	Interleukin-13
IL-17	Interleukin-17
ILD	Interstitial lung disease
IPF	Idiopathic pulmonary fibrosis
IP3R	Inositol 1,4,5-trisphosphate recepto
JAK2	Janus kinase 2
JNK	c-Jun N-terminal kinase
LKB1	Liver kinase B1
lncRNA	Long non-coding RNA
LOX	Lysyl hydroxylase
MASH	Metabolic Dysfunction-Associated Steatohepatitis
MMP9	Matrix metalloproteinase 9
MAPK	Mitogen-activated protein kinase
MCP-1	Monocyte chemoattractant protein-1
MESA	Multi-Ethnic Study of Atherosclerosis
miR-29	MicroRNA-29
miR-148b-3p	microRNA-148b-3p
MyD88	Myeloid differentiation primary response 88
NADPH	Nicotinamide adenine dinucleotide phosphate
NAFLD	Non-alcoholic fatty liver disease
NF-κB	Nuclear factor kappa B
NFATc	Nuclear factor of activated T cells, cytoplasmic
NLRP3	NOD-, LRR- and pyrin domain-containing protein 3
NO	Nitric oxide
NOG	NOD/Shi-scid/IL-2Rγnull
OVA	Ovalbumin
p38	p38 mitogen-activated protein kinase
PAI-1	Plasminogen activator inhibitor-1
PASMC	Pulmonary artery smooth muscle cell
PBMC	Peripheral blood mononuclear cell
PH	Pulmonary hypertension
PI3K	Phosphoinositide 3-kinase
PKA	Protein kinase A
PLC	Phospholipase C
PPAR-γ	Peroxisome proliferator-activated receptor gamma
RAGE	Receptor for advanced glycation end products
RELM	Resistin-like molecule
*RELMα*	Resistin-like molecule alpha
*FIZZ2*	Resistin-like molecule beta
*Retnlα*	Resistin-like molecule alpha
RETN	Human resistin gene
RETNLB	Human resistin-like beta gene
Retn	Mouse resistin gene
*Retnla*	Mouse resistin-like alpha gene
*Retnlb*	Mouse resistin-like beta gene
*Retnlg*	Mouse resistin-like gamma gene
RGD	Arginine-glycine-aspartic acid
ROR1	Receptor tyrosine kinase-like orphan receptor 1
ROS	Reactive oxygen species
RP-ILD	Rapidly progressive interstitial lung disease
Sp1	Specificity protein 1
SDF-1	Stromal cell-derived factor 1
SERCA2a	Sarco/endoplasmic reticulum Ca^2+^-ATPase 2a
Sirt1	Sirtuin 1
Smad3	Smad family member 3
SOCS3	Suppressor of cytokine signaling 3
SOCE	Store-operated calcium entry
SSc	Systemic sclerosis
STAT3	Signal transducer and activator of transcription 3
STAT6	Signal transducer and activator of transcription 6
STIM1	Stromal interaction molecule 1
TAC	Transverse aortic constriction
T2DM	Type 2 diabetes mellitus
TGF-β1	Transforming growth factor-beta 1
Th2	T helper 2
TIRAP	Toll/interleukin-1 receptor domain-containing adaptor protein
TLR4	Toll-like receptor 4
TNF-α	Tumor necrosis factor alpha
TZDs	Thiazolidinediones
VEGF	Vascular endothelial growth factor
VSMCs	Vascular smooth muscle cells
α-SMA	Alpha-smooth muscle actin
β-MHC	Beta-myosin heavy chain
ΔDCN	Delta-decorin
